# Comparative IgG-IgM Western Blot in the Diagnosis of Congenital Toxoplasmosis: A Valid Diagnostic Tool

**DOI:** 10.3390/pathogens15020225

**Published:** 2026-02-17

**Authors:** Alice Bonetti, Ambra Vola, Daniele Lilleri, Lucrezia Lo Grasso, Milena Furione, Alessia Arossa, Alessandra Ricciardi, Claudia Viganò, Alessia Bressan, Claudia Pavia, Annalisa De Silvestri, Giulia Gambini, Fausto Baldanti, Valeria Meroni

**Affiliations:** 1 Microbiology and Virology Unit, Diagnostic Medicine Department, Fondazione IRCCS Policlinico San Matteo, 27100 Pavia, Italy; 2 PhD National Programme in One Health Approaches to Infectious Diseases and Life Science Research, Department of Public Health, Experimental and Forensic Medicine, University of Pavia, 27100 Pavia, Italy; 3 Department of Obstetrics and Gynecology, Fondazione IRCCS Policlinico San Matteo, 27100 Pavia, Italy; 4 Infectious Diseases Unit, Fondazione IRCCS Policlinico San Matteo, 27100 Pavia, Italy; 5 Neonatalogy and Neonatal Intensive Care Unit, Fondazione IRCCS Policlinico San Matteo, 27100 Pavia, Italy; 6 Microbiology Unit, Department of Laboratory Medicine and Diagnostic Biotechnology, Azienda Socio Sanitaria Territoriale (ASST) Ovest Milanese, Hospital of Legnano, 20025 Milan, Italy; 7 Scientific Direction, Clinical Epidemiology and Biometric Unit, Fondazione IRCCS Policlinico San Matteo, 27100 Pavia, Italy; 8 Department of Clinical, Surgical, Diagnostic and Pediatric Sciences, University of Pavia, 27100 Pavia, Italy; 9 Department of Molecular Medicine, University of Pavia, 27100 Pavia, Italy

**Keywords:** IgM ISAGA, comparative western blot, congenital toxoplasmosis

## Abstract

Congenital toxoplasmosis (CT) results from vertical transmission of *Toxoplasma gondii* during maternal infection in pregnancy. Early diagnosis in newborns is crucial to initiate timely therapy and prevent long-term sequelae. The IgM Immunosorbent Agglutination Assay (ISAGA) has historically been considered an important diagnostic tool for CT; however, its recent market withdrawal necessitates alternative approaches. We conducted a retrospective observational study at Fondazione IRCCS Policlinico San Matteo, Pavia, Italy, including 44 newborns born to mothers with confirmed toxoplasmosis between 2019 and 2022. Newborns were classified as CT (n = 19) or non-CT (n = 25) based on serological follow-up, comparative Western blot (CWB) and Interferon Gamma Release Assay (IGRA). Sensitivity and specificity of CWB, IgM Chemiluminescent Immunoassay (CLIA), and IgM ISAGA were assessed at birth and at one month. At birth, CWB demonstrated 88.9% sensitivity, significantly higher than IgM CLIA (52.6%) and IgM ISAGA (57.9%). Specificity was 100% at birth and 92% at one month. CWB retained high sensitivity at one month (81.8%). IGRA complemented CWB in confirming or excluding infection in cases with equivocal or false-negative serology. Comparative Western blot thus represents a robust diagnostic alternative for CT, ensuring early detection and timely treatment, particularly in the absence of IgM ISAGA.

## 1. Introduction

Toxoplasmosis is a systemic infection caused by *Toxoplasma gondii* (Nicolle & Manceaux, 1908), a protozoan parasite classified within the phylum Apicomplexa. This microorganism has an exceptionally broad host range, infecting various species, including birds and both aquatic and terrestrial mammals, humans among them. In individuals with a competent immune system, *Toxoplasma gondii* infection is typically asymptomatic and self-resolving, as it is effectively controlled by both cellular and humoral immune responses [1].

Congenital toxoplasmosis (CT) occurs when *T. gondii* is vertically transmitted to the fetus from a mother who acquired the primary infection during pregnancy or shortly before.

According to the literature, the risk of fetal infection increases with gestational age: at 6, 18, and 30 weeks of pregnancy, the risk of embryonic/fetal infection is reported as 2.2%, 23.0%, and 56.0% [2], respectively. However, it has been proven that prompt treatment of the newly born and multidisciplinary management can help reduce these percentages [3]. On the other hand, the severity of disease in the fetus is inversely proportional to the time of maternal infection. Although the likelihood of transmission is higher in late gestation, infections acquired earlier in pregnancy are generally associated with more severe clinical outcomes in the fetus, including neurological and ocular damage. Therefore, the timing of maternal infection is a critical factor both for transmission probability and for the severity of fetal disease.

The consequences of fetal infection vary and may include spontaneous abortion, stillbirth, permanent neurological or visual sequelae, as well as completely asymptomatic infections. The clinical presentation of congenital toxoplasmosis depends on several factors, including the virulence of the Toxoplasma gondii strain involved, and the parasitic load transmitted to the fetus.

Regarding prenatal diagnosis by Polymerase Chain Reaction (PCR) on amniotic fluid, this is a sensitive and specific technique; however, it is not always performed, making highly sensitive tests in the newborn necessary [3].

Early diagnosis of CT is crucial to enable the timely initiation of appropriate therapy, which helps prevent long-term damage that could affect the child’s development. Therefore, identifying the infection in newborns in the first months of life is essential for effective medical intervention [4].

One of the investigated markers of CT is IgM in the serum of newborns, as IgM does not cross the placenta and therefore indicates a fetal immune response.

In the diagnostic algorithm of CT, the IgM ISAGA (BioMérieux, Marcy-l’Étoile, France) test has played a crucial role, due to its high sensitivity and specificity. However, due to changes in European in vitro diagnostic regulations, the production and marketing of IgM ISAGA have been stopped in 2024. This removal posed a significant challenge in diagnosing CT, as a positive IgM ISAGA result is considered a sensitive and specific marker of the disease [5,6,7].

The aim of this retrospective observational study is to assess an alternative diagnostic algorithm, designed to determine the sensitivity and specificity of the comparative Western blot (CWB–LBDIO Diagnostic, Lyon, France) in the diagnosis of congenital toxoplasmosis.

## 2. Materials and Methods

This retrospective observational study was conducted in an Italian referral hospital: Fondazione IRCCS Policlinico San Matteo, Pavia.

At Fondazione IRCCS Policlinico San Matteo, a dedicated multidisciplinary team manages infections during pregnancy, from laboratory diagnosis to the clinical care of at-risk pregnancies and clinical/serological follow-up of newborns.

The study included 44 newborns who were referred to our center and were born to mothers with documented *T. gondii* infection during pregnancy between 2019 and 2022; for some of these mothers, the pregnancies had been managed at other centers. In accordance with Italian guidelines, pregnant women diagnosed with toxoplasmosis received spiramycin (3 million units, three times daily) as first-line therapy until delivery. When seroconversion occurred after the 21st week of gestation, treatment was switched to a combination of pyrimethamine–sulfadiazine with folinic acid. The pyrimethamine–sulfadiazine regimen was also initiated in cases of positive *T. gondii* PCR on amniotic fluid; in these situations, spiramycin was temporarily discontinued and subsequently reintroduced as monotherapy approximately three weeks prior to delivery.

Only newborns with available one-year follow-up data were included to confirm or exclude congenital infection.

At birth, each infant underwent a comprehensive clinical and neurological evaluation, together with targeted serological testing. Ocular involvement was assessed by direct and indirect dilated fundoscopy to identify chorioretinitis or other toxoplasmosis-related lesions, while transfontanellar ultrasound was performed to rule out ventricular dilatation or intracranial calcifications.

Serological testing was performed monthly during the first three months of life and subsequently at two-month intervals until one year of age.

Newborns were classified into two groups: (i) with congenital toxoplasmosis (CT) and (ii) without congenital toxoplasmosis (Non-congenital Toxoplasmosis—NCT).

The absence of congenital infection was confirmed by the negativization of specific IgG antibodies within the first year of life in the absence of therapy. Conversely, an increase in IgG levels or the emergence of IgM and/or IgA antibodies indicates congenital infection.

According to the international and Italian guidelines [2,8,9], diagnosis of congenital toxoplasmosis was established if at least one of the following conditions was present:

- Positive IgM or IgA for *Toxoplasma gondii*;
- IgG/IgM synthesized by newborn, identified through CWB;
- IgG rebound and/or persistence at 12 months of age;
- Positive Interferon Gamma Release Assay (IGRA) as confirmatory test.

In case of confirmed CT, treatment of infected newborns with pyrimethamine-sulfadiazine was promptly started.

The following exams were performed on each sample:

- VIDAS^®^ Toxo IgG II (BioMérieux, Marcy-l’Étoile, France);
- CLIA LIAISON XL^®^ Toxo IgG (DiaSorin, Saluggia, Italia);
- CLIA LIAISON XL^®^ Toxo IgM (DiaSorin, Saluggia, Italia);
- IgM ISAGA (BioMérieux, Marcy-l’Étoile, France);
- IgA Enzyme-Linked Immunosorbent Assay (ELISA–NovaLisa, Dietzenbach, Germany)
- Comparative Western blot (CWB–LBDIO Diagnostic, Lyon, France) for mother/newborn at birth or newborn/newborn at the second control (LBDIO Diagnostic, Lyon, France);
- Interferon-Gamma Release Assay (IGRA in-house test) [10].

All congenital infections were confirmed by a positive IGRA test and by the presence of IgG antibodies at the one-year follow-up.

All tests were performed following the manufacturer’s Instructions for Use (IFUs) [11,12], and the WB was carried out in a semi-automated manner.

**Figure 1:**
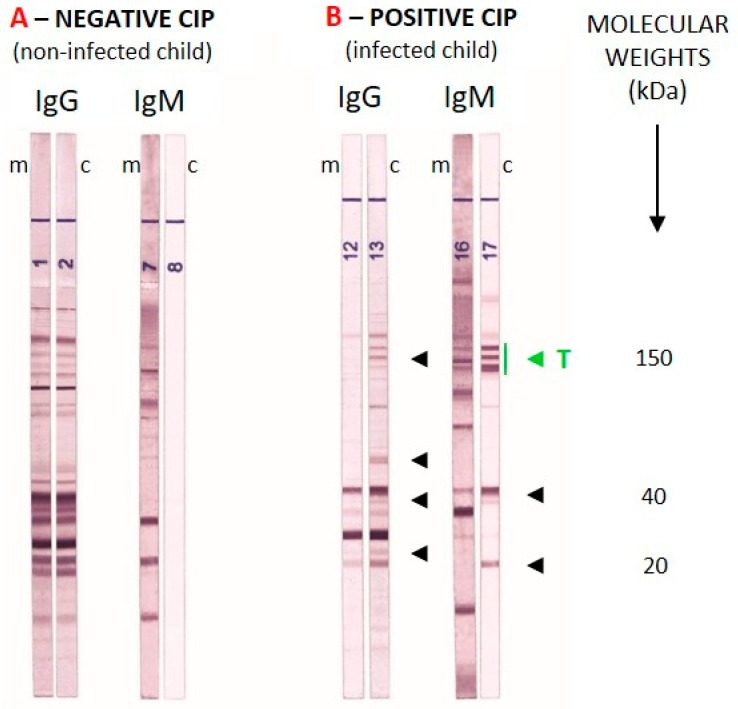
On the left, an example of comparative Western blot in a non-congenital infection. On the right, an example of congenital toxoplasmosis diagnosed by comparative Western blot; arrows indicate additional bands present in the newborn’s serum. A classic IgM pattern is a triplet of bands at 75, 90, and 100 kDa (see green “T” in the image); m, mother; c, child. Image taken from the test kit’s Instructions for Use [12].

Any well-defined band with a molecular weight between 20 kDa and <120 kDa was analyzed visually by comparing maternal and neonatal samples collected at birth. For the IgG strip, any additional band present in the neonatal serum but absent in the maternal serum was considered positive. Because IgM does not cross the placenta and therefore reflects a fetal immune response, any IgM band detected in the neonatal serum was considered positive. The same criteria were applied at the one-month follow-up, with comparisons made between the two neonatal sera (at birth and at one month). An example of comparative WB analysis for a NCT and a CT newborn is shown in Figure 1.

The study obtained ethical approval from the Ethics Committee of Fondazione IRCCS Policlinico San Matteo (protocol number 0012545125).

## 3. Results

A total of 44 serum samples from 44 newborns were tested at birth: 25 cases of NCT (56.8%) and 19 of CT (43.2%). Data concerning mothers with an acute *T. gondii* infection during pregnancy were available for 43 patients (97.7%), as one case in the CT group involved an abandoned infant for whom no maternal sample was available.

Within the NCT group, each newborn was tested twice: at birth and again one month later. Among the CT cases, all the 19 newborns were tested at birth, but only 11 newborns (57.9%) were tested at both time points.

All newborns from the NCT group exhibited IgG CLIA and IgG ELFA positivity at birth (100.0%), with 2/25 (8.0%) showing IgG negativization at the time of the second sampling. No cases of IgM positivity were recorded by IgM CLIA or ISAGA, nor was any comparative CWB IgG positivity observed at birth (0%). However, 2/25 (8.0%) infants presented equivocal CWB IgM findings at one-month follow-up. In both cases, a negative IGRA test excluded congenital infection. Thus, the overall specificity of the comparative Western blot was 100% at birth and 92% at the one-month follow-up.

Within the CT group, all newborns showed IgG positivity by both CLIA and ELFA at birth (100.0%). Eight out of 19 newborns (42.1%) tested positive for IgM by CLIA, 2/19 (10.5%) showed an equivocal IgM CLIA result, while 11 out of 19 (57.9%) were positive by IgM ISAGA. Only 2 out of 18 infants (11.1%) had completely negative IgG and IgM WB results, whereas the remaining newborns displayed various combinations of IgG and/or IgM reactivity, ranging from positive to equivocal. Accordingly, the overall sensitivity of the CWB was 88.9% (Table 1 and Table 2). Comparison using McNemar’s test showed that Western blot IgG/IgM had significantly higher sensitivity in detecting positive cases at birth compared to both IgM CLIA (*p* = 0.008) and IgM ISAGA (*p* = 0.014).

At one month follow-up, 1 newborn out of the 11 CT newborns examined (9.1%) tested positive for IgM by CLIA, 1/11 (9.1%) showed an equivocal IgM CLIA result, while 5 out of 11 (45.5%) were positive by IgM ISAGA.

Two out of 11 infants (18.2%) had negative CWB results for both IgG and IgM (these two infants were already negative at birth), whereas the remaining newborns displayed various combinations of IgG and/or IgM reactivity, ranging from positive to equivocal. (Table 3 and Table 4)

Accordingly, the overall sensitivity of the CWB at one month was 81.8%. Comparison using McNemar’s test showed that Western blot IgG/IgM had significantly higher sensitivity in detecting positive cases at birth compared to both IgM CLIA (*p* = 0.014) and IgM ISAGA (*p* = 0.046).

## 4. Discussion

The IgM ISAGA test has long represented a diagnostic cornerstone of congenital toxoplasmosis (CT) due to its sensitivity and specificity. Its withdrawal from the market has caused a significant diagnostic gap, prompting a search for alternative diagnostic algorithms. IgM ISAGA has high sensitivity, ease of use, and low cost, and as such has been adopted for routine diagnostics across many laboratories [5,6,7].

The comparative Western blot (CWB) assay also demonstrates excellent diagnostic performance, and our study highlights its very high sensitivity at birth, which makes it a reliable tool for the early identification of congenital infection. The observed reduction in sensitivity at one month of life should not be considered an intrinsic limitation of the method, but rather the expected consequence of the prompt initiation of pyrimethamine–sulfadiazine therapy in congenitally infected newborns, which impairs antibody production.

As a referral center for the diagnosis of toxoplasmosis, Fondazione IRCCS Policlinico San Matteo in Pavia routinely uses comparative WB testing, which has proven to be a valid alternative to the discontinued IgM ISAGA. Comparative evaluation in both uninfected neonates and CT cases confirmed the excellent specificity of WB at birth (100.0%), with only a minor decrease at one month (92.0%) due to equivocal IgM results. The high sensitivity and specificity of this test allow for the prompt initiation of treatment in newborns with congenital infection, preventing delays that could lead to poor clinical outcomes.

It is also important to highlight the crucial role of in house IGRA test in excluding congenital infection in two cases with equivocal Western blot and in confirming it in four cases with false-negative IgM ISAGA and Western blot. The implementation of a diagnostic algorithm including Western blot and IGRA test in reference laboratories could further reduce the clinical impact of congenital toxoplasmosis by shortening the time to diagnosis and enabling earlier initiation of therapy in infected newborns.

This study has some limitations that should be acknowledged. The sample size was relatively small, reflecting the limited number of confirmed cases of congenital toxoplasmosis with complete follow-up. In addition, some heterogeneity in treatment and follow-up protocols may have occurred, as patients were managed in different clinical settings, reflecting real-life practice.

In conclusion, comparative Western blot represents a valuable diagnostic tool that appears fundamental in the algorithm for congenital toxoplasmosis, particularly in the context of IgM ISAGA commercial discontinuation. Its main limitations remain the relatively high costs, the need for trained personnel for both performance and interpretation, and the requirement for paired maternal–infant samples.

## Figures and Tables

**Table 1:** IgM and Western blot Results at Birth in the CT group (N = 19).

Test (at Birth)	Positive	Equivocal	Negative	Positive/Equivocal (%)
IgM CLIA	8	2	9	52.6
IgM ISAGA	11	0	8	57.9
Western blot IgG/IgM ^1^	16		2	88.9

^1^ One newborn was not tested by Western blot because the mother sample was not available. CWB (Western blot IgG/IgM) results included various combinations of IgG and/or IgM reactivity in 16 infants; 2 newborns had completely negative results.

**Table 2:** Comparative Western blot at birth, CT group (N = 18).

IgG	IgM	N	%
Pos	Pos	6	33.3
Pos	Eqv	1	5.6
Pos	Neg	2	11.1
Neg	Pos	2	11.1
Neg	Eqv	1	5.6
Eqv	Pos	3	16.7
Eqv	Eqv	1	5.6
Eqv	Neg	0	0
Neg	Neg	2	11.1

Results of heterogeneous patterns of IgG and/or IgM reactivity in the CT group, with results classified as negative (Neg), equivocal (Eqv), or positive (Pos).

**Table 3:** IgM and Western blot Results at one-month follow-up (N = 11).

Test (at birth)	Positive	Equivocal	Negative	Positive/Equivocal (%)
IgM CLIA	1	1	9	18.2
IgM ISAGA	5	0	6	45.5
Western blot IgG/IgM ^1^	9		2	81.8

^1^ CWB (Western blot IgG/IgM) results included various combinations of IgG and/or IgM reactivity in 9 infants; 2 newborns had completely negative results.

**Table 4:** Comparative Western blot at birth, CT group (N = 11).

IgG	IgM	N	%
Pos	Pos	2	18.2
Pos	Eqv	0	0
Pos	Neg	1	9.0
Neg	Pos	0	0
Neg	Eqv	0	0
Eqv	Pos	0	0
Eqv	Eqv	1	9.0
Eqv	Neg	5	45.5
Neg	Neg	2	18.2

Results of heterogeneous patterns of IgG and/or IgM reactivity in the CT group, with results classified as negative (Neg), equivocal (Eqv), or positive (Pos).

## Data Availability

The data that support the findings of this study are available on request from the corresponding author. The data are not publicly available due to privacy or ethical restrictions.

## Abbreviations

The following abbreviations are used in this manuscript:

CT	Congenital toxoplasmosis
ISAGA	Immunosorbent Agglutination Assay
CLIA	Chemiluminescent Immunoassay
PCR	Polymerase Chain Reaction
CWB	Comparative Western Blot
NCT	Non-congenital Toxoplasmosis
IGRA	Interferon Gamma Release Assay
ICU	Instructions for Use
